# Supplementary material for: Induction of osteogenesis by bone-targeted Notch activation
Source: eLife. 2022 Feb 4;11:e60183. doi: 10.7554/eLife.60183 (PMC8880996; doi:10.7554/eLife.60183)
Supplement: Figure 1—figure supplement 1—source data 2. [file elife-60183-fig1-figsupp1-data2.pdf]

A

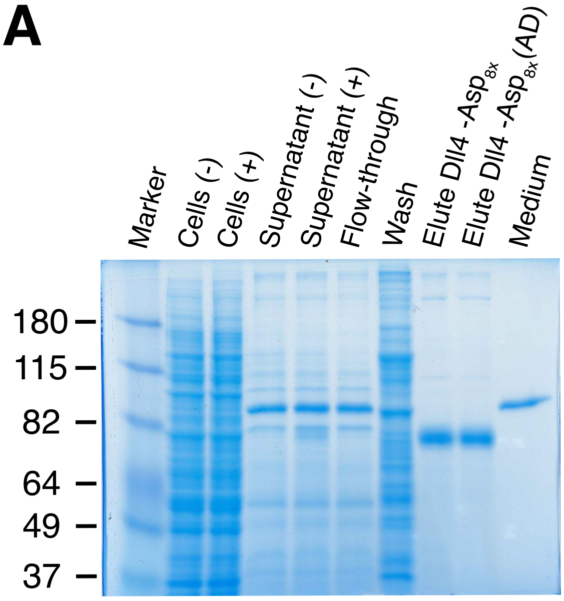

Xu, Dinh et al., Figure 1-figure supplement 1A

B

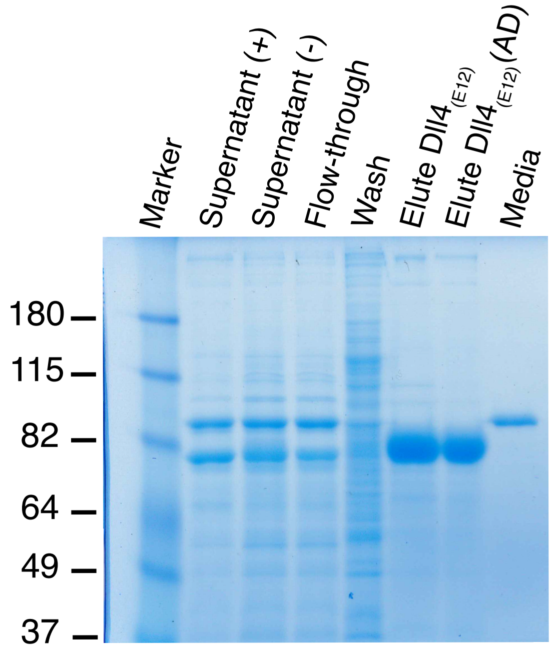

Xu, Dinh et al., Figure 1-figure supplement 4B

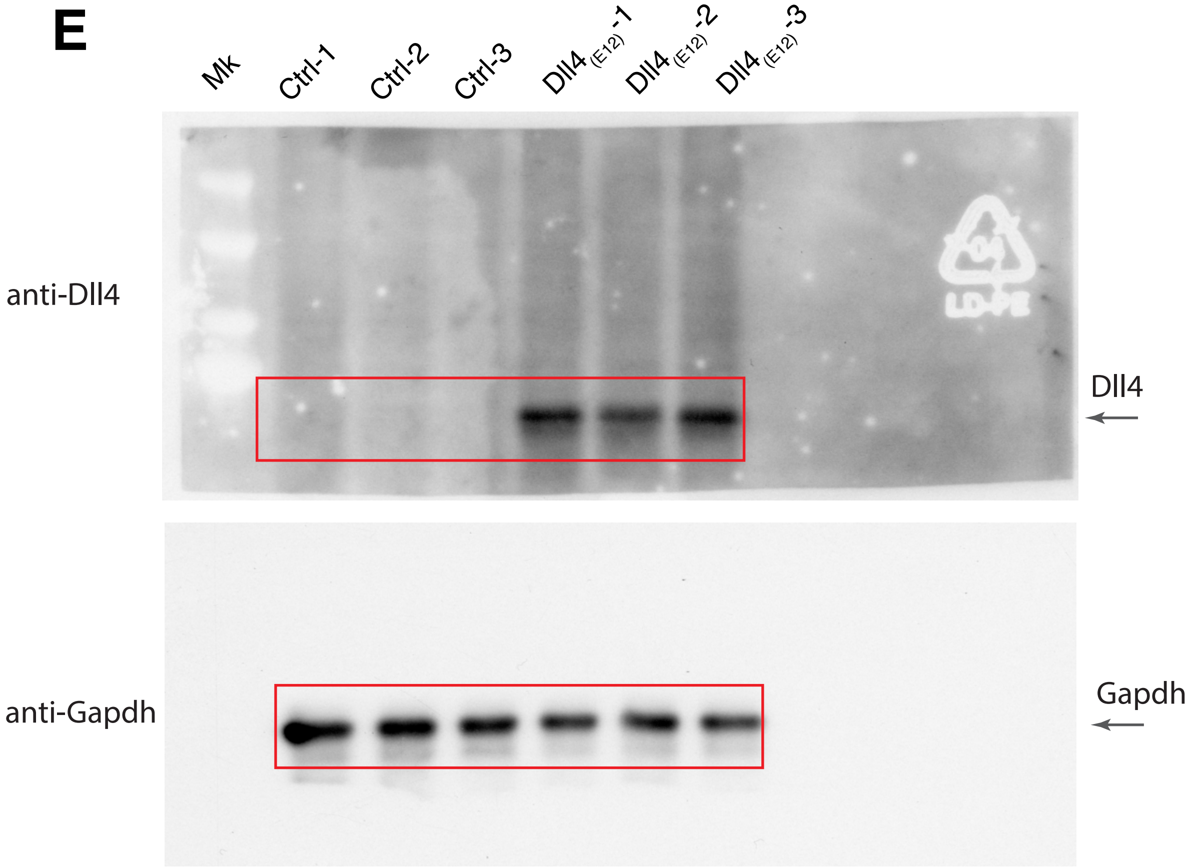

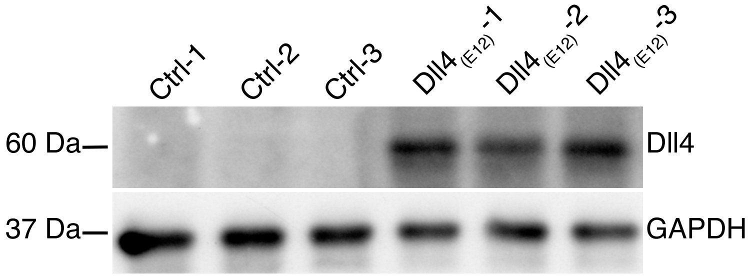

Xu, Dinh et al., Figure 1-figure supplement 4E

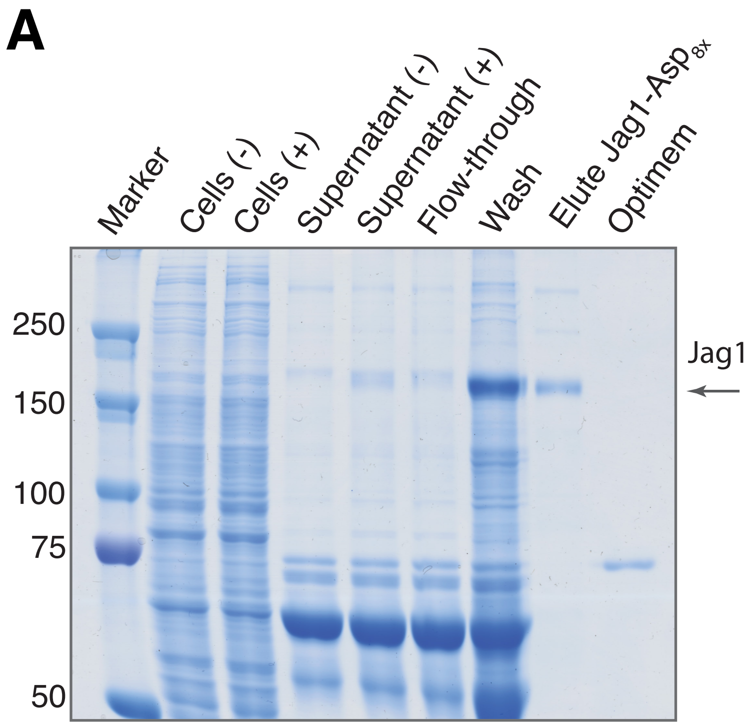

Xu, Dinh et al., Figure 5-figure supplement 1A

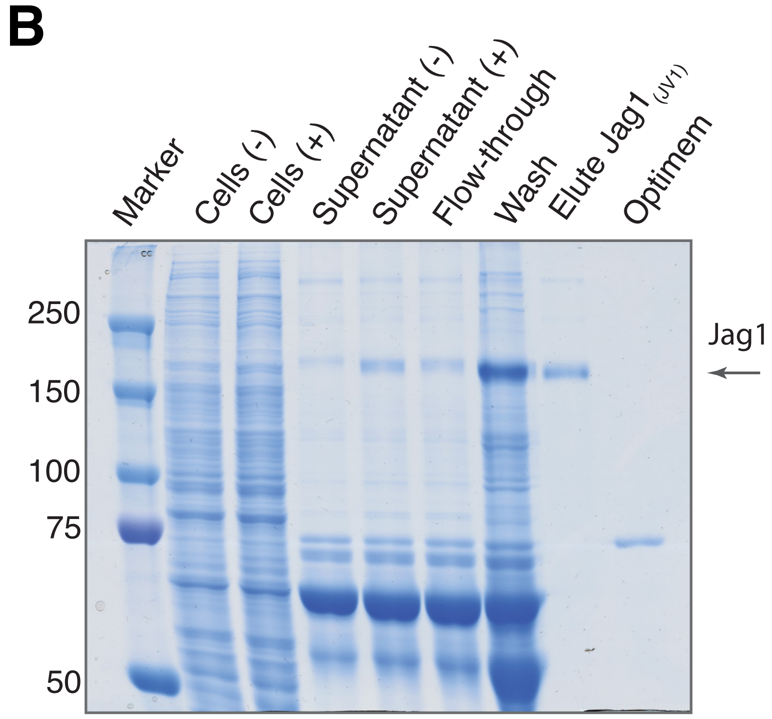

Xu, Dinh et al., Figure 5-figure supplement 1B

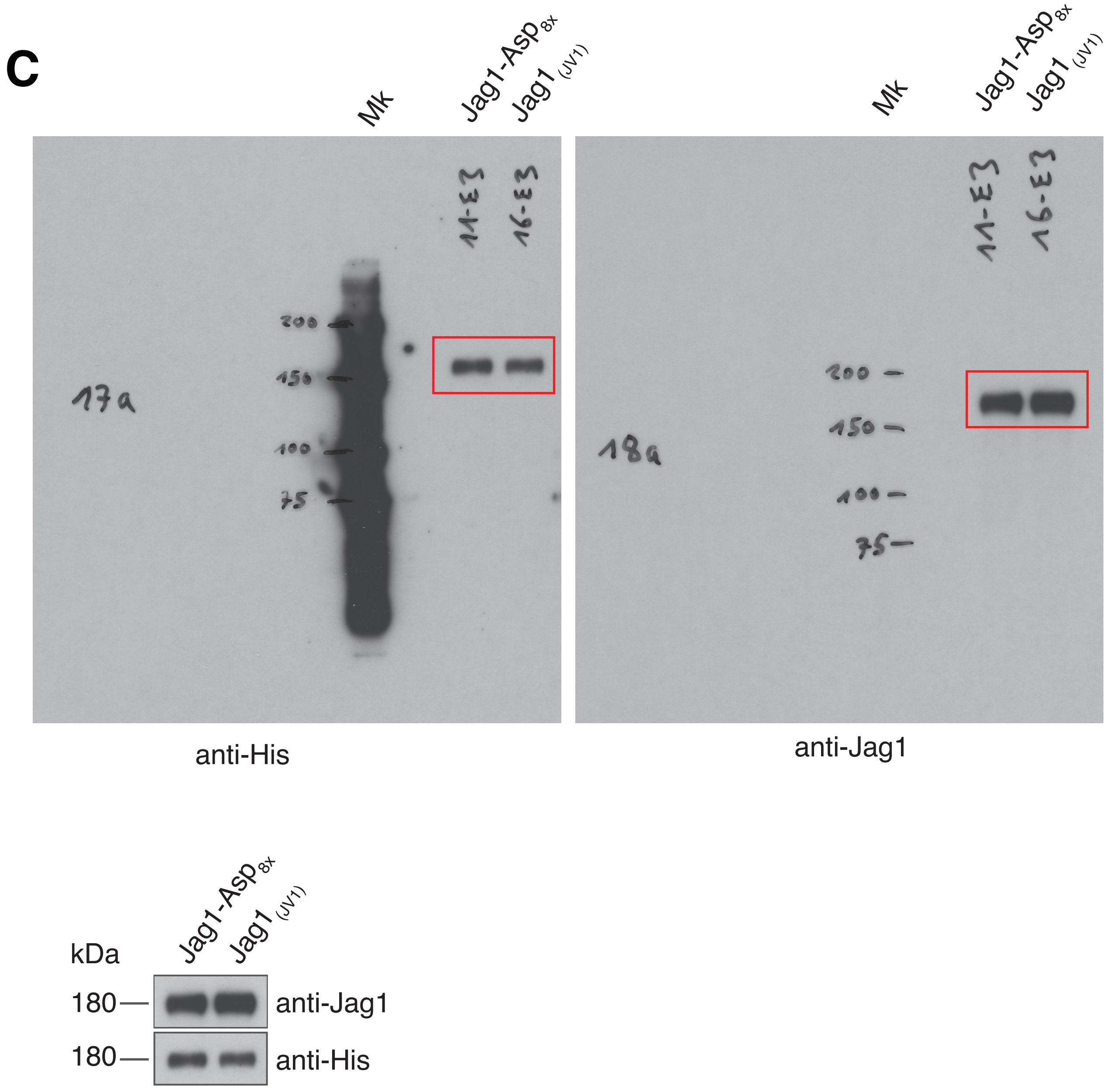

Xu, Dinh et al., Figure 5-figure supplement 1C
