# Supplementary figures and images for: Induction of osteogenesis by bone-targeted Notch activation
Source: eLife. 2022 Feb 4;11:e60183. doi: 10.7554/eLife.60183 (PMC8880996; doi:10.7554/eLife.60183)

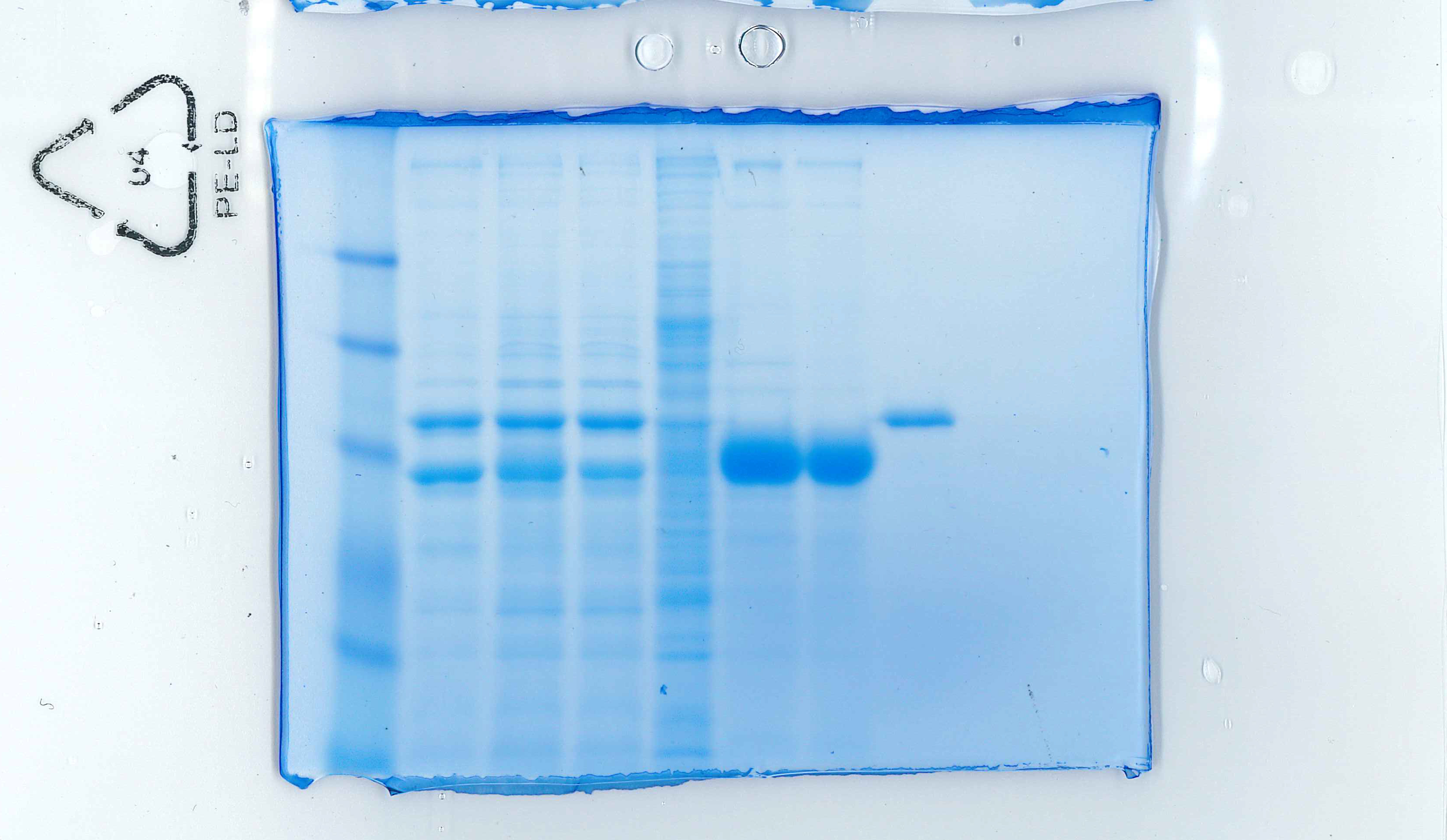

Supplement: Figure 1—figure supplement 1—source data 3. [file elife-60183-fig1-figsupp1-data3.zip › Figure 1-figure supplement 4B-source data-4B-original.jpg]

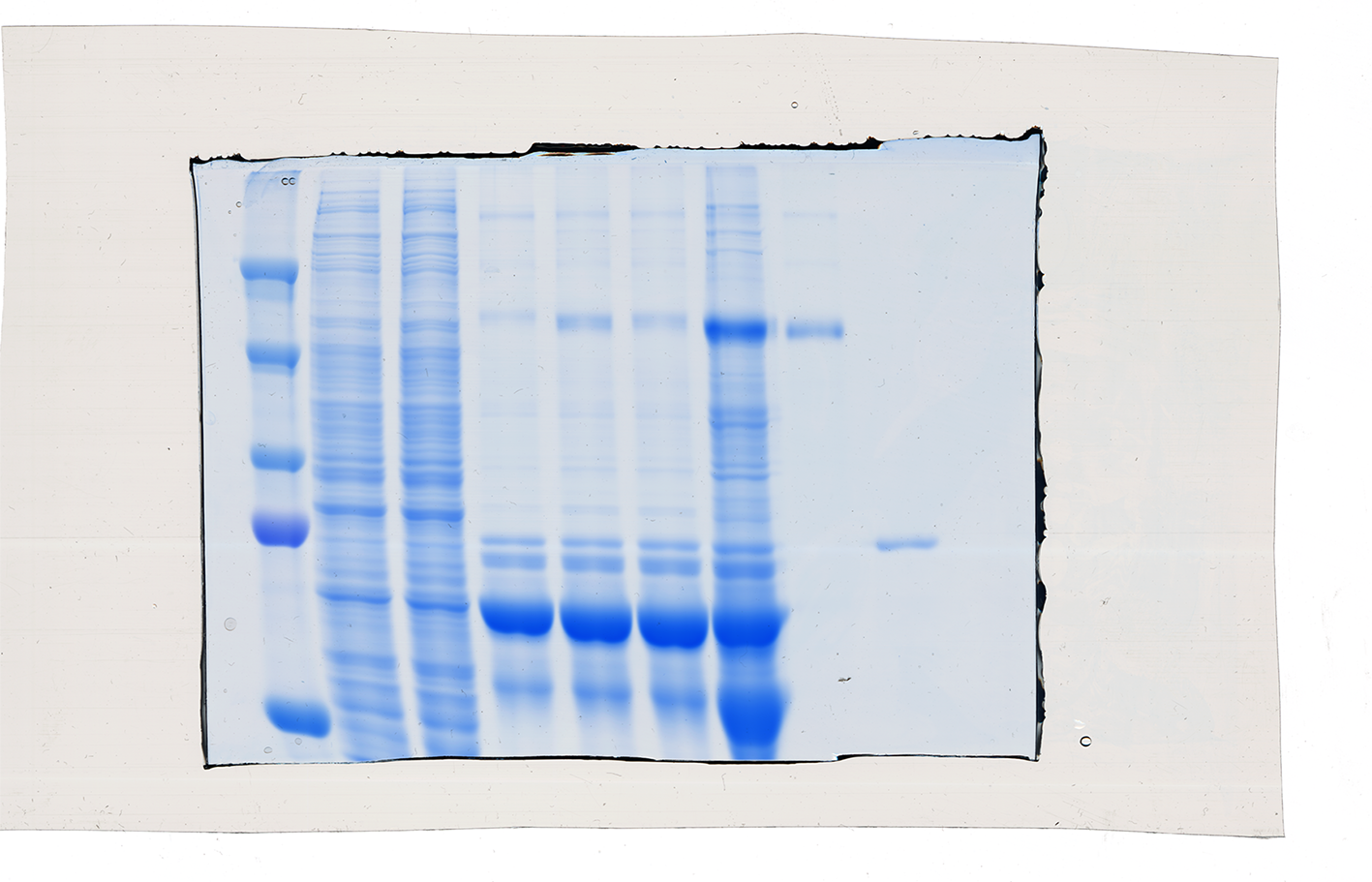

Supplement: Figure 1—figure supplement 1—source data 3. [file elife-60183-fig1-figsupp1-data3.zip › Figure 5-figure supplement 1B- source data 1B_original.tif]

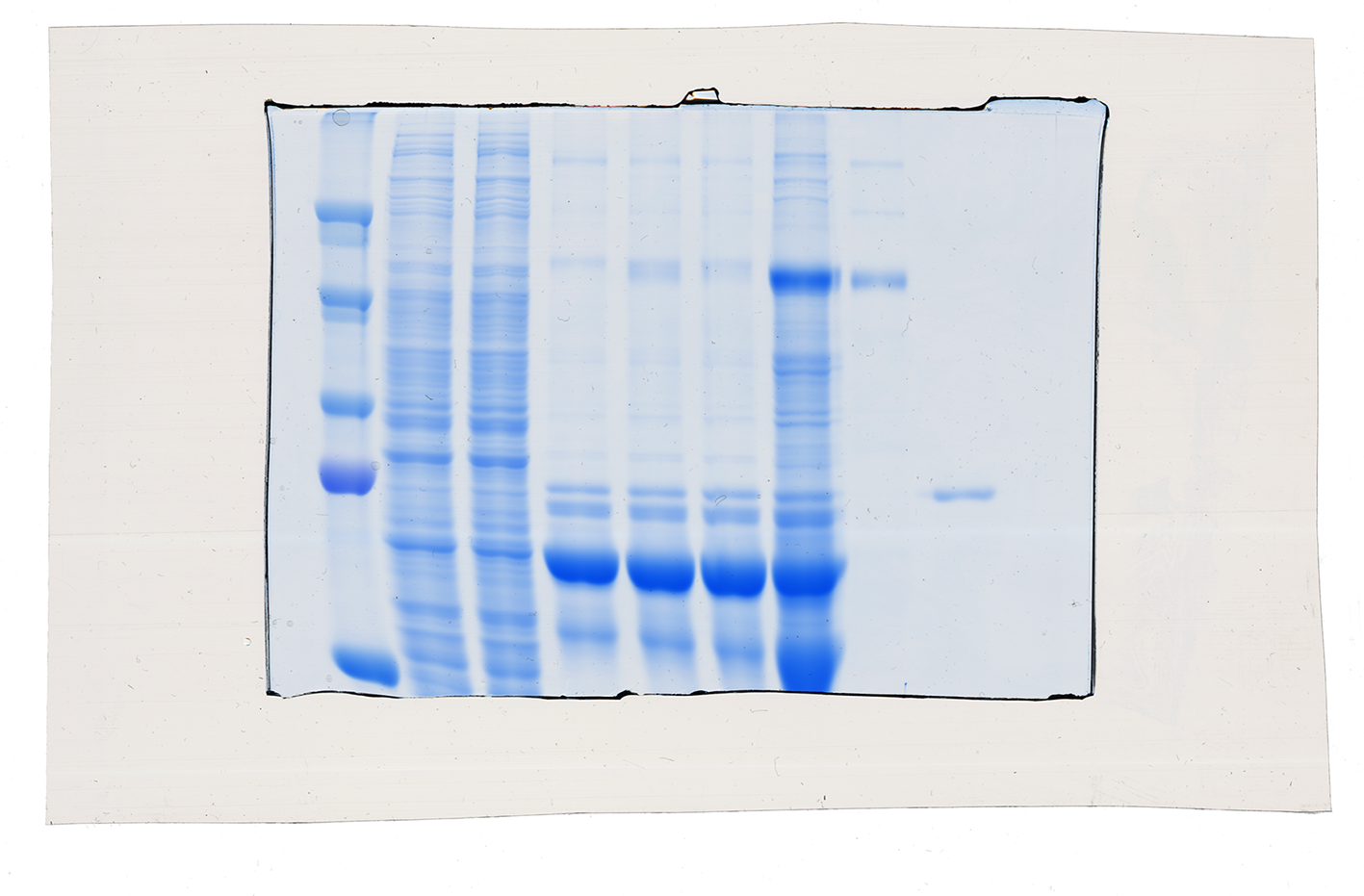

Supplement: Figure 1—figure supplement 1—source data 3. [file elife-60183-fig1-figsupp1-data3.zip › Figure 5-figure supplement 1A- source data 1A_original.tif]

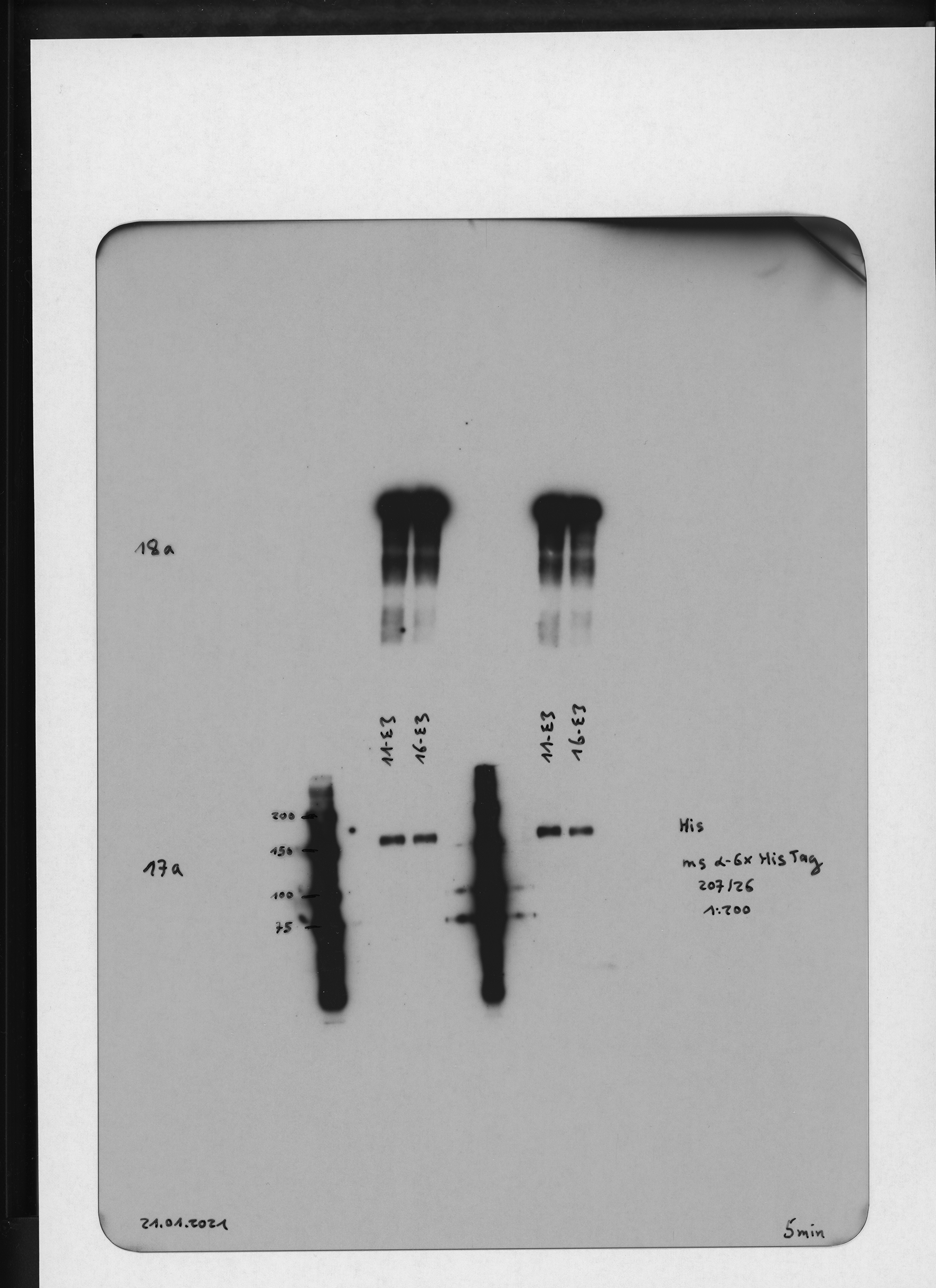

Supplement: Figure 1—figure supplement 1—source data 3. [file elife-60183-fig1-figsupp1-data3.zip › Figure 5-figure supplement 1C- source data 1C_anti-His_whole_blot_original.tif]

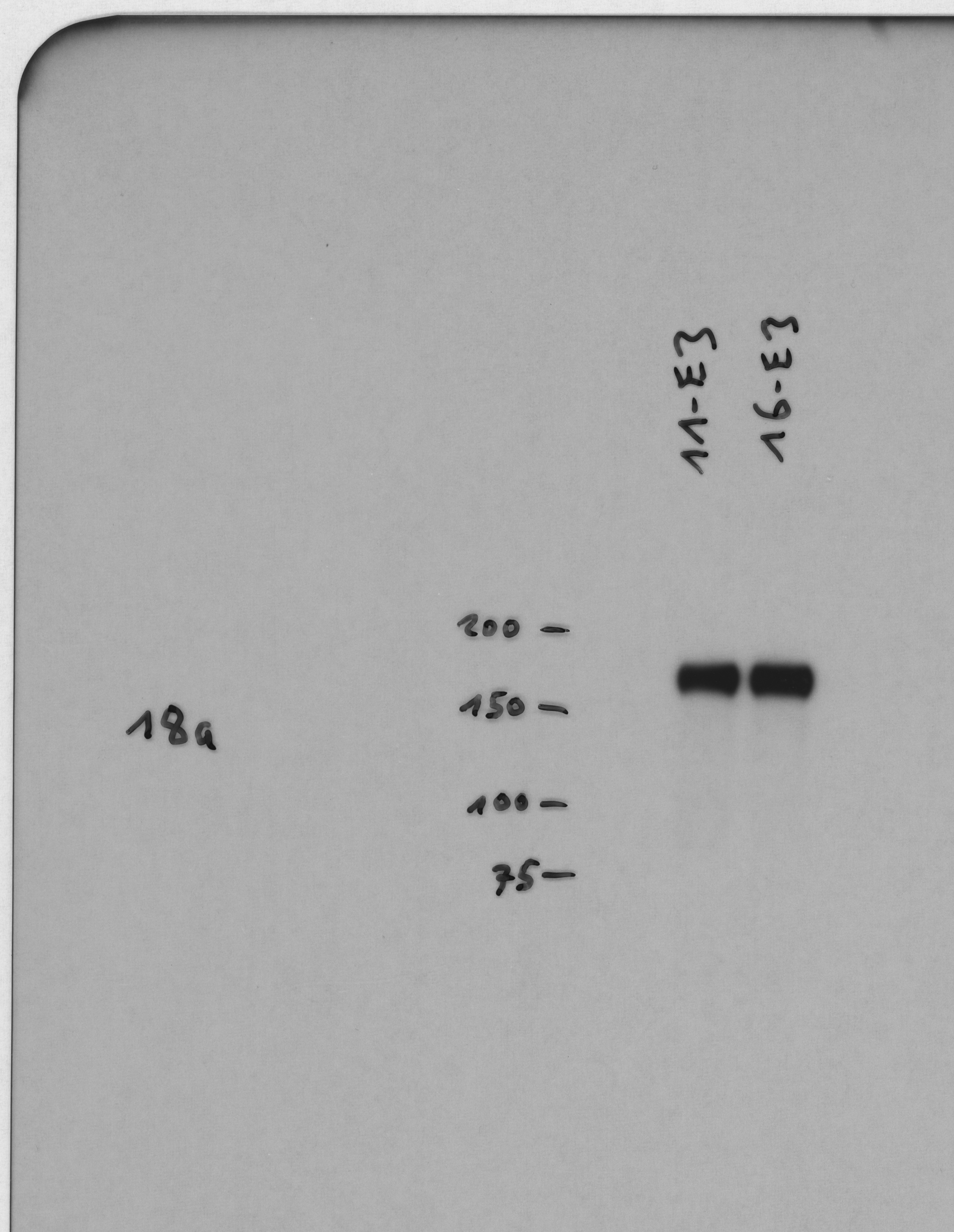

Supplement: Figure 1—figure supplement 1—source data 3. [file elife-60183-fig1-figsupp1-data3.zip › Figure 5-figure supplement 1C- source data 1C_anti-Jag1_original.tif]

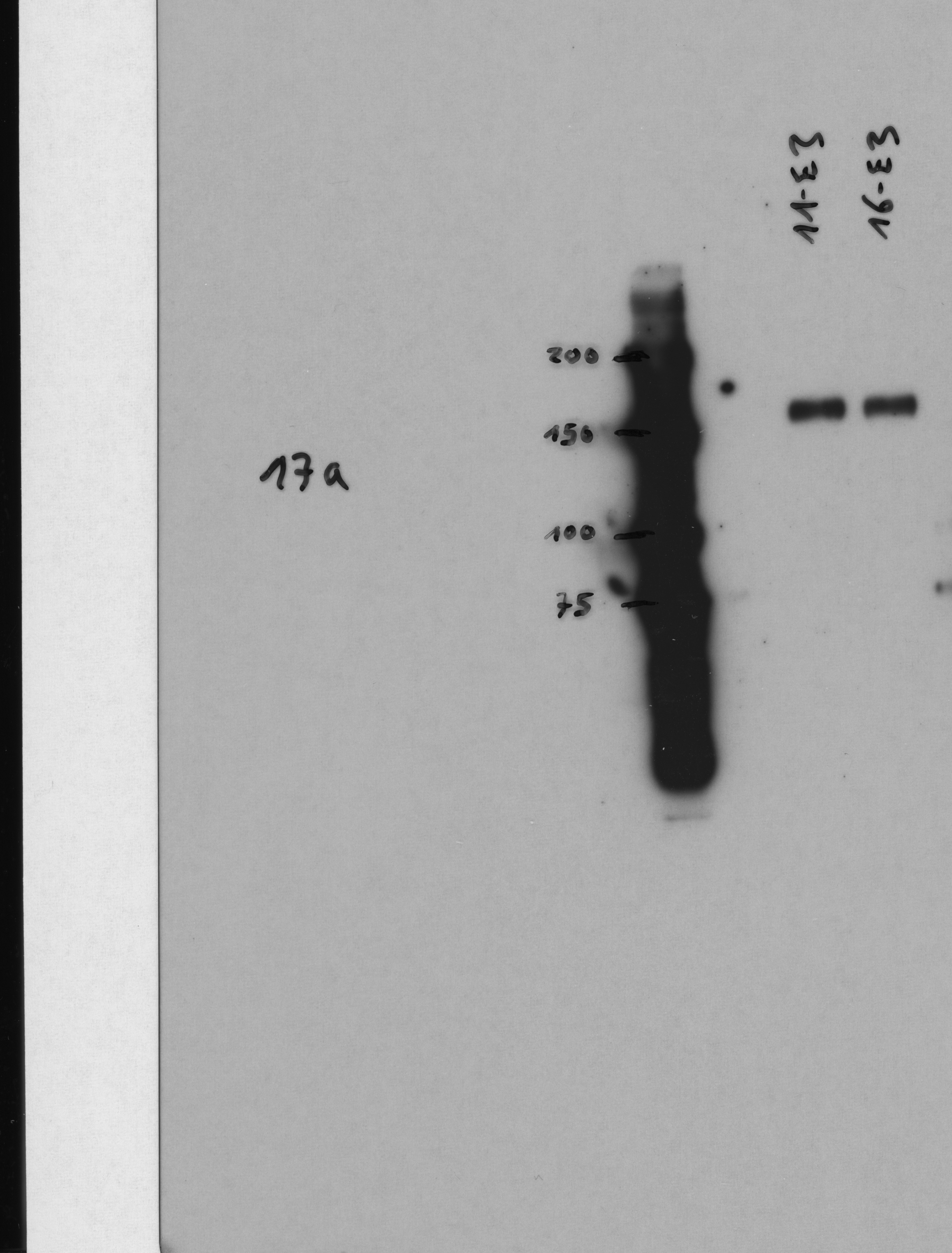

Supplement: Figure 1—figure supplement 1—source data 3. [file elife-60183-fig1-figsupp1-data3.zip › Figure 5-figure supplement 1C- source data 1C_anti-His_original.tif]

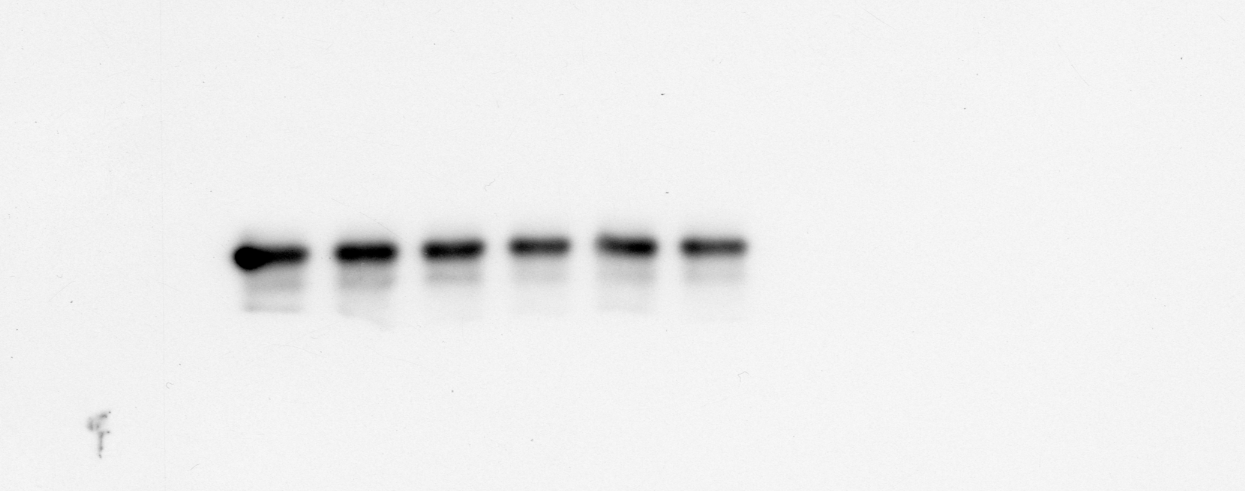

Supplement: Figure 1—figure supplement 1—source data 3. [file elife-60183-fig1-figsupp1-data3.zip › Figure 1-figure supplement 4E- source data_anti-Gapdh_gel_original.png]

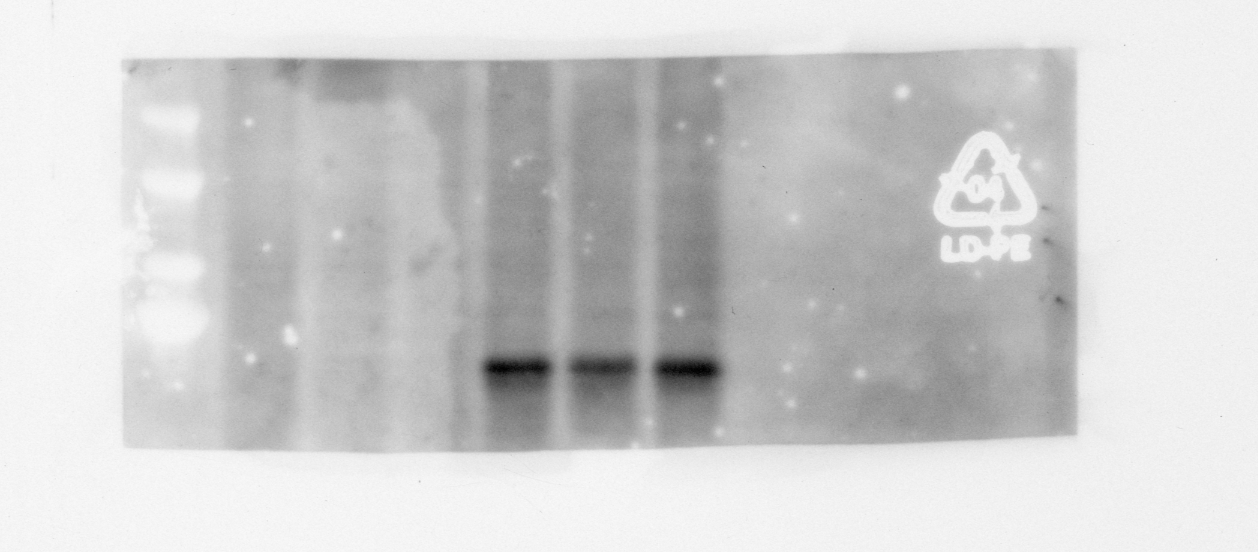

Supplement: Figure 1—figure supplement 1—source data 3. [file elife-60183-fig1-figsupp1-data3.zip › Figure 1-figure supplement 4E- source data_anti-Dll4_gel_original.png]

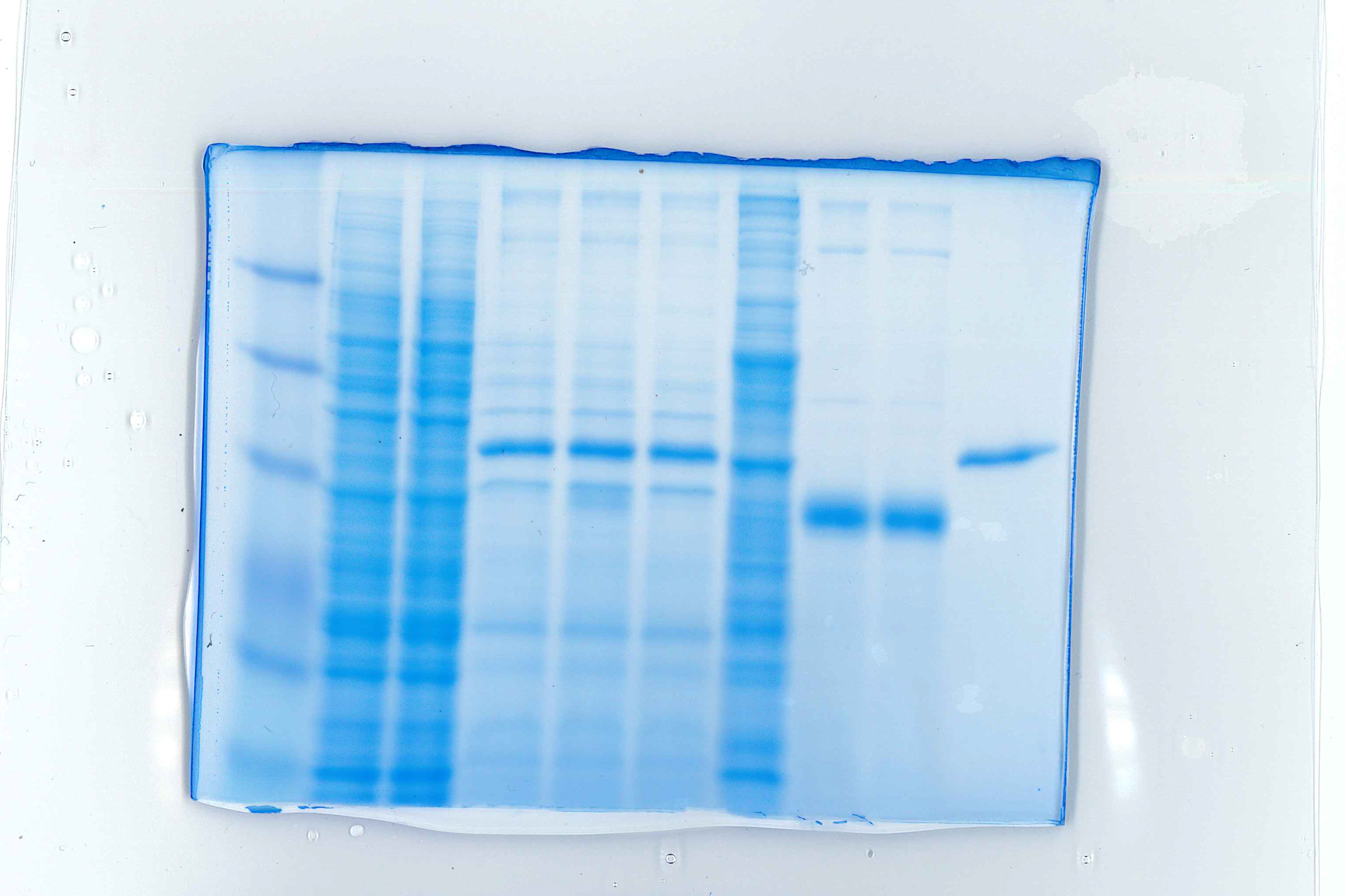

Supplement: Figure 1—figure supplement 1—source data 3. [file elife-60183-fig1-figsupp1-data3.zip › Figure 1-figure supplement 1A_source data-1A-original.jpg]
